# Assessing the Value of Antibiotics on Farms: Modeling the Impact of Antibiotics and Vaccines for Managing *Lawsonia intracellularis* in Hog Production

**DOI:** 10.3389/fvets.2019.00364

**Published:** 2019-10-18

**Authors:** Travis Jansen, Alfons Weersink, Michael von Massow, Zvonomir Poljak

**Affiliations:** ^1^Department of Food, Agricultural and Resource Economics, University of Guelph, Guelph, ON, Canada; ^2^Department of Population Medicine, Ontario Veterinary College, University of Guelph, Guelph, ON, Canada

**Keywords:** antibiotic resistance, cost-effectiveness, simulation model, *Lawsonia intracellularis*, preventative treatment

## Abstract

Increasing awareness of antibiotic resistance has correspondingly increased efforts to identify and reduce the causal behaviors that led to this severe public health threat. The consequences of these efforts are regulatory and market pressures limiting antibiotic use by livestock farmers which may lead to significant financial and welfare challenges on the farm, even if antibiotics can be substituted by vaccines. The purpose of this study is to measure the relative cost-effectiveness of antibiotics vs. vaccines for controlling *L. intracellularis* on a Canadian farrow-to-finish pig farm. This is done by modeling the production and economic impact of different antibiotics and vaccines available for managing this disease, listed in the Canadian Compendium of Veterinary Products. The economic impacts (in Canadian dollars) of the disease are estimated and the net benefits of alternative prevention and treatment options are compared to determine the relative cost-effectiveness of each strategy. Of the 12 options analyzed, four were preventative (antibiotic and vaccine) and eight were antibiotic treatments. Prophylactic chlortetracycline (an antibiotic) is the most cost-effective option for managing *L. intracellularis*, while Porcilis Ileitis (a vaccine) is the least cost-effective strategy. This result remains robust considering sensitivity analysis of the production parameters, which indicates that preventative antibiotics are more cost-effective than vaccines. This implies that banning preventative antibiotic treatments harms the bottom line of farmers under current market conditions.

## Introduction

Antibiotic resistance is one of the most significant threats facing human and animal healthcare systems today (1). Less than a century after discovering penicillin, overuse and misuse of antibiotics has increased the rate that antibiotic-resistant bacteria develop, rendering some antibiotics ineffective. With very few synthetic antibiotics being developed and no effective substitutes available, maintaining the efficacy of existing antibiotics is vital for the continued success of all healthcare systems (2).

Given the significant volume of antibiotics administered to livestock, many consider reducing animal antibiotics as one way to reduce antibiotic resistance in humans (1). In an effort to reduce livestock antibiotic use, the Canadian government has prohibited the use of antibiotics for growth promotion since December of 2018 (3). At this time, the government also required that farmers in all provinces have a veterinary prescription for the antibiotics that they use. These rules brought consistent regulation across the country in an effort to improve farmers' relationships with veterinarians and increase prudent antibiotic use. These rules reflect the government's efforts to reduce antibiotic resistance on livestock farms in Canada, but it is unknown how they will impact farmers' ability to run a profitable business.

Antibiotics provide economic benefits to farmers by promoting growth, preventing diseases, and treating the diseases that do arise in their livestock. Economic research on antibiotic use has tended to focus on measuring the value of antibiotic growth promoters (AGPs) (4, 5). Similar to the industry's current situation, these studies emerged as a result of increasing evidence that AGPs were unnecessary and contributed to the development of antibiotic resistant bacteria (6). Without AGPs, there are unanswered questions surrounding the cost-effectiveness of the remaining antibiotics and how they can be used, particularly the financial impact to farmers if the ban is extended to other antibiotics.

The monetary losses from not being able to use antibiotics for disease management could be offset depending on the efficacy and price of vaccines. Vaccines are preventative in nature as they provide a non-threatening exposure of a specific disease to an animal that prepares the animal's immune system to fight off the disease once infected. Because vaccines are simply priming the body's immune system, they have the potential to reduce the clinical impact of specific diseases without contributing to antibiotic resistance. Unfortunately, vaccines are typically disease-specific and do not offer the broad effects of many antibiotics, which can increase the cost and number of products necessary for disease prevention. Furthermore, vaccines must typically be injected, which requires more labor than using preventive antibiotics that are added to the feed or water. While there are studies showing an economic benefit to vaccinating for diseases like *Mycoplasma hyopneumoniae* (7) and Circovirus Type 2 (8), there is little research comparing the financial benefit to vaccines as compared to preventive antibiotics.

Given that the impact of reduced antibiotic use on livestock farms is not known, the purpose of this study is to demonstrate how the relative cost-effectiveness of antibiotics and vaccines can be measured for managing diseases in livestock. A bioeconomic farm enterprise modeling framework was created to measure these differences. This article uses this framework to model the production and financial impacts of *Lawsonia intracellularis* (*L. intracellularis*) in pigs at the farm level, with and without intervention from vaccine and antibiotic options…Antibiotics and vaccines are the focus of this study because these are the products that are used for disease intervention. While management options such as improved biosecurity will reduce the occurrence of disease (thus reducing the need for both products), biosecurity is for disease prevention and not disease intervention or management. An empirical model of a representative farrow-to-finish pig farm in Canada is developed and the costs of the disease without intervention are initially established. The reduction in these costs from alternative antibiotic and vaccine strategies are then estimated. The identification of the cost-effective management options to deal with *L. intracellularis* can help farmers, veterinarians, and government representatives to reduce antibiotic use while maintaining the financial viability of the farm. Similar adapted modeling frameworks can also be used to evaluate the net financial benefits that preventive and treatment antibiotics and vaccines provide to farms with other livestock and other diseases and can also be extended to consider the economic impacts beyond the farm gate.

## Materials and Methods

### Overview of the Model

**Figure 1:**
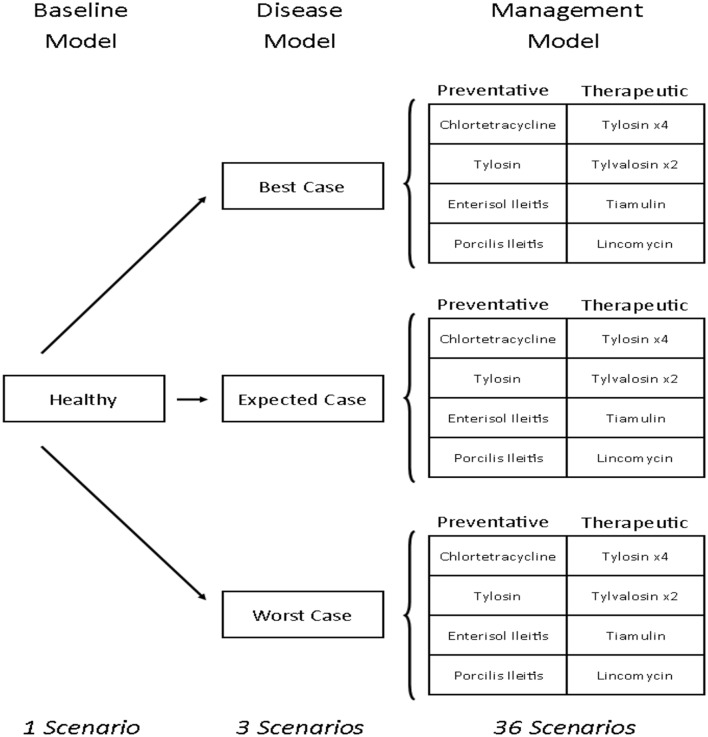
Pig barn scenarios. Tylosin ×4 indicates that there are four different treatment options that use tylosin as the antibiotic. Tylvalosin ×2 indicates two different treatment options that use tylvalosin as the antibiotic.

This study develops a bioeconomic modeling framework that can be used to measure the financial impacts of antibiotics and vaccines for managing disease in livestock. Specifically, this is done by estimating and comparing the relative cost-effectiveness of 12 antibiotic and vaccine options for managing *L. intracellularis* on a typical Canadian farrow-to-finish swine farm. To do this, the production and profitability (in Canadian dollars) of a pig farm were calculated under 40 different farm scenarios (Figure 1). The first scenario is the baseline empirical model and reflects a typical Canadian farrow-to-finish swine farm under normal conditions. The next three are disease scenarios which were created to reflect the different severity levels associated with the occurrence of *L. intracellularis*. Twelve disease management strategies were then imposed on each of the three disease scenarios with four considered as preventative and the other eight as therapeutic. The net result was 40 scenarios: one baseline with no disease, three disease scenarios with no treatment, and 36 disease management scenarios (12 for each of the three disease severity assumptions). The cost of *L. intracellularis* can be estimated by comparing the profits with and without the disease and the cost-effective management options can be determined by comparing profitability across the treatments under a given disease scenario.

One of the advantages of this bioeconomic simulation model is that it can adapt to limited representative data availability, as is the case in some of the 12 different treatments examined on swine farms, by focussing on the relative ranking of the treatments, not necessarily their absolute values. Variability in production and economic information can also be addressed by checking the robustness of the rankings using sensitivity analysis. Combined with the thorough accounting of the modeling parameters presented in this article, the model outputs can be compared and contrasted to any farm situation, although they are not intended to represent any particular farm.

The first part of the following section outlines the methods used to build the baseline empirical model. The second part provides the assumptions on the productivity and mortality impacts of the disease scenarios while the third part discusses the efficacy of the management options within each disease scenario.

### Baseline Model

A static and deterministic empirical model was created in Excel® to represent the operations of a typical farrow-to-finish operation in Canada. This type of model was deemed appropriate for this research given the endemic nature of *L. intracellularis* in pig herds.

In Canada, pig farms are generally separated into three different production stages: the sow barn, the nursery barn and the finishing barn. The sow barn is where the breeding animals are housed, and piglets are nursed. In this model, the breeding cycle involves 2 days of breeding, a 115-day gestation period, 21 days of nursing and then a 5-day break before beginning the cycle again. At 21 days, piglets are weaned and sent to the nursery barn. They spend 8 weeks in this barn before being moved to a finishing barn. Here, they will spend 16 weeks growing before they leave the farm and are shipped to a processing plant. Cull sows are assumed to be sold and replacement gilts are assumed to be purchased in.

In the sow barn, mature female pigs are housed to raise their piglets. Assuming 21 days for weaning, a 5-day weaning to breeding interval, 2 days of breeding and a 115-day gestation length, a sow can produce 2.55 litters per year. For a 1,000 head sow-herd that batch-farrows weekly, this results in 20 groups of sows with 50 sows per group. Assuming the farmer sells his sows as culls after the sixth parity and there is 0.8% sow herd mortality per parity, the replacement rate for sows is 17.5% per parity or 44.62%, annually.

Therefore, in each group, 41 of the females are sows and 9 of the females are gilts. If sows wean 11.31 piglets and gilts wean 9.35 piglets per breeding, then, annually, this combines to 27.96 pigs/sow/year. Assuming an 85% farrowing rate, this amounts to ~24,239 pigs per year being shipped to the nursery barn, or 466 pigs per week. Pigs are then kept in the nursery for 8 weeks and, with typical mortality around 3%, there are ~452 pigs being shipped from the nursery to the finishing barn per week for a loss of about 14 pigs per group. Provided there is 2% mortality in the finishing barn over a 16-week period, this amounts to 443 pigs being shipped per week to the processing plant, or about 23,042 pigs per year. The production parameters in this model are created from the parameters used by Weng et al. (9) and through discussions with swine experts at the University of Guelph, including active swine farmers. A summary of the production parameters used in the baseline model can be found in Table 1.

**Table 1:** Production parameters of farrow-to-finish swine farm.

**Parameter**	**Value**	**Source**
**BREEDING HERD NUMBERS**		
**Breeding Cycle (Days)**		
Wean-breed interval	5	(9)
Breeding time	2	(9)
Gestation length	115	Author's assumption
Nursing period	21	Author's assumption
Total cycle length	**143**	
Days per year	365	
Cycles per year	**2.55**	
**Breeding Groups**		
Farrowing per year (weekly)	52	
Cycles per year	2.55	
Number of breeding groups	**20**	
Size of herd	1,000	
Size of breeding group	**50**	
**Sow Replacement Per Parity**		
<6 parity sow herd mortality	0.8%	Author's assumption
Sows >6 Parity	16.7%	Author's assumption
Replacement rate	**17.5%**	
**Group Breakdown**		
Sows per group	50	
Gilts per group	9	
>1 parity sows per group	41	
**Sow Performance**		
Farrowing rate	85%	(9)
**Gilts**		
Gilts successfully bred	7.4	
Born alive	11	(9)
Preweaning litter mortality	15%	(9)
Weaned per gilt	**9.4**	
**>1 Parity Sows**		
>1 parity sows successfully bred	34.7	
Born alive	13	Author's assumption
Preweaning litter mortality	13%	(9)
Weaned per >1 parity sows	**11.3**	
Piglets sent to nursery per week	**466**	
**Nursery Barn Numbers**		
Pigs entering per week	466	
Weeks in nursery	8	Author's assumption
Barn mortality	3%	Author's assumption
Total nursery inventory	3660.2	
Piglets moved to finishing barn per week	**452.2**	
**Finishing Barn Numbers**		
Pigs entering per week	452.2	
Weeks in finisher	16	Author's assumption
Barn mortality	2%	Author's assumption
Total finisher inventory	7,157.6	
Market pigs shipped for processing per week	**443.1**	
Market pigs shipped for processing per year	**23,041.7**	

The 443 hogs shipped per week are assumed to be sold at the average price of $3.39 per kg and a dressed weight that is 80% of live weight. The average monthly hog price in Ontario has been normally distributed since January 2000 and the assumed market price is the average monthly price over this period. The hog index is assumed to be 110.00, which is the value used by the Ontario Ministry of Agriculture, Food and Rural Affairs (OMAFRA) in their Swine Enterprise Budgets since 2014 (OMAFRA various) (10).

The cost of growing hogs includes the feed, other variable costs and fixed costs. Feed represents approximately two-thirds of total production costs for a marketed pig. Feed consumption is based on the feed conversions and weight gain values listed in Table 2. These values are the authors' estimations based on experience within the Canadian swine industry, including on active farm operations. Feed costs are calculated based on this consumption and ration prices that were provided from a local feed industry expert (source asked to remain anonymous due to competition with other suppliers). Other variable costs and fixed costs come from the OMAFRA Swine Enterprise Budget and are an average from January 2000 and November 2017. Other variable costs include veterinary costs, breeding supplies, marketing, grading, trucking, utilities, miscellaneous, repairs, maintenance, labor, and operating loan interest. Fixed costs include depreciation, interest, taxes, and insurance.

**Table 2:** Feed and weight assumptions for growing pigs.

	**Weight (kg)**	**Feed conversion**	**Average daily gain (kg)**
**Weaning**	6.00		
**Nursery Barn**			
Week 4	7.51	1.30	0.22
Week 5	9.64	1.30	0.31
Week 6	12.41	1.30	0.40
Week 7	15.99	1.35	0.51
Week 8	20.40	1.40	0.63
Week 9	24.92	1.45	0.65
Week 10	29.54	1.55	0.66
Week 11	34.16	1.70	0.66
**Finishing Barn**			
Week 12	38.66	2.07	0.64
Week 13	43.70	2.34	0.72
Week 14	48.74	2.34	0.72
Week 15	54.82	2.43	0.87
Week 16	60.90	2.43	0.87
Week 17	67.20	2.88	0.90
Week 18	73.50	2.88	0.90
Week 19	80.43	3.06	0.99
Week 20	87.36	3.06	0.99
Week 21	94.29	3.24	0.99
Week 22	101.22	3.24	0.99
Week 23	108.15	3.60	0.99
Week 24	115.08	3.60	0.99
Week 25	122.01	3.60	0.99
Week 26	128.94	3.42	0.99
Week 27	135.87	3.42	0.99
**Shipping**	135.87		

*Parameter values were guided by http://www.thepigsite.com/stockstds/17/growth-rate/ and adjusted based on consultations with swine researchers at the University of Guelph*.

In the baseline model, sows are assumed to eat two rations, a lactation feed and a dry feed. For 21 days of lactation, sows are assumed to eat 6.37 kg of feed per day at a cost of $0.32/kg or $315.40/ton. For the remaining 122 days, sows are assumed to eat, on average, 2.55 kg of dry feed per day at a cost of $0.26/kg or $263.71/ton. The total cost to feed one sow for the year is $316.22 or $13.72 per marketed pig. This model assumes all gilts are bought in and the net cost for replacing cull sows is $70. The cost for gilts to replace deceased sows is $130 plus the estimated value of a market pig. At baseline, this cost is estimated to be about $2.01 per marketed pig. In the sow barn, other variable costs are $18.07 per marketed pig and fixed costs are $7.23 per marketed pig.

Nursery piglets are assumed to only eat one ration for the 8-week period at a cost of $348.24/ton. Over the 8 weeks, pigs are assumed to eat 40.91 kg of feed for a cost of $16.19 per marketed pig. In the nursery barn, other variable costs are $6.50 per marketed pig and fixed costs are $3.74 per marketed pig. In the finishing barn, pigs are assumed to eat two rations, the grower ration ($290.43/ton) for the first 7 weeks and a finisher ration ($264.29/ton) for the final 9 weeks. Over the 16 weeks in the finishing barn, pigs are assumed to eat a total of 285.96 kg of feed for a cost of $85.42 per pig shipped. In the finishing barn, other variable costs are $14.19 per marketed pig and fixed costs are $12.18 per marketed pig.

The net result per marketed pig for the baseline is revenue of $183.82 and total costs of $178.83 for a profit of $4.99, which is typical of industry average conditions for Ontario and consequently serves as one means of validating the model.

### Disease Scenarios

Three scenarios are created to describe the severity of clinical signs from *L. intracellularis*, which is the typical approach to categorize disease impacts (11, 12). Note that we are assuming the occurrence of the disease as we are assessing the antibiotic and vaccine treatment options as opposed to controlling the disease specifically. The effects of the disease on mortality and feed efficiency are summarized for each of the three categories in Table 3.

**Table 3:** Production impact of *L. intracellularis* on a 1,000-sow farrow-to-finish hog farm.

		***Lawsonia intracellularis*** **disease scenario**		
**Production parameter**	**Baseline-healthy**	**Best-case**	**Expected**	**Worst-case**
Length of clinical signs^a^^,^ ^b^	–	2 weeks	5 weeks	9 weeks
Nursery barn mortality^c^	3%	4%	6%	8%
Average daily gain^b^	(see Table 2)	↓6%	↓13%	↓20%
Feed conversion ratio^b^	(see Table 2)	↑6%	↑15%	↑25%

*Disease scenarios were created by the author based on data from the following sources*.

a *Stege et al. (13)*.

b *McOrist and Gebhart (14)*.

c *Winkelman (15)*.

For fattening pigs, the clinical signs from *L. intracellularis* generally appear in the nursery barn at about 8–10 weeks of age but may show up anywhere between 6 and 20 weeks of age (13, 14). Herd mortality increases slightly with *L. intracellularis*, ranging anywhere from 1 to 5% (15). The major damage caused by *L. intracellularis* comes from its impact on pig growth. Because *L. intracellularis* colonize the epithelial cells of the small intestine, this causes a thickening of the gut wall which can reduce the ability of the pig to digest food and absorb nutrients (16, 17). McOrist and Gebhart (14) report that average weight gains are reduced by 6–20% in infected pigs and feed conversion decreases by 6–25%.

Three disease scenarios were created using the above information. The best-case scenario assumes the lowest estimated impact: mortality increases to 4% in the nursery barn, average daily gain falls by 6% and an additional 6% more feed is required for the same amount of weight gain. In the best-case scenario, these problems are assumed to persist for 2 weeks if no intervention strategy is taken. The expected scenario assumes that over the next 5 weeks mortality increases to 6% in the nursery, average daily gain falls by 13% and feed efficiency declines by 15%. In the worst-case scenario, mortality in the nursery reaches 8%, average daily gain falls by 20% and an additional 25% more feed is required for the same amount of weight gain. In the worst-case scenario, problems continue for 9 weeks, provided there is no intervention by the farmer. In all three scenarios, clinical signs begin to appear in the pigs at 10 weeks of age.

### Methods for Managing *Lawsonia intracellularis*

The Canadian Edition of the Compendium of Veterinary Products lists 12 options for managing *L. intracellularis*. These options consist of five antibiotics and two vaccines. However, several antibiotics can be administered in different combinations and/or rates and these variations allow for 10 antibiotic management options from the five antibiotics.

The 12 management options, their doses and associated costs are listed in Table 4. Four of these options are preventative management options and can be used pre-emptively to reduce the severity of production problems once a pig is introduced to *L. intracellularis*. Two of these options are antibiotics, chlortetracycline and tylosin, and two are vaccines, Enterisol Ileitis and Porcilis Ileitis. Chlortetracycline and tylosin are provided in the feed of nursery pigs. For chlortetracycline, it is suggested that it is put in the feed of pigs for the 2 weeks prior to an expected outbreak. If tylosin is used, then it is to be put in the feed for 3 weeks prior to the expected outbreak. However, due to the significant health challenges that recently weaned pigs face, antibiotics, like chlortetracycline and tylosin, tend to be given to pigs as soon as they enter the nursery. In an attempt to represent true farm scenarios, it is assumed preventative chlortetracycline or tylosin are given as soon as the pigs enter the nursery barn.

**Table 4:** *L. intracellularis* management strategies listed in the Canadian compendium of veterinary products.

**Option**		**Antibiotic or vaccine**	**Initial age for treatment**	**Length of treatment**	**Given via**	**Dose**	**Cost ($/gm or $/ml)**	**Cost ($/pig)**
**PREVENTATIVE MANAGEMENT**								
1	Chlortetracycline	Antibiotic	3 weeks	14 days	Feed	22 mg per kg of body weight	$1.49	$0.25
2	Tylosin	Antibiotic	3 weeks	21 days	Feed	110 g per kg of feed	$0.26	$0.24
3	Enterisol Ileitis	Vaccine	3 weeks	Once	Drench	2 mL	$0.68	$1.36
4	Porcilis Ileitis	Vaccine	3 weeks	Once	Injection	2 mL	$0.69	$1.38
**TREATMENT MANAGEMENT**								
5	Tylosin	Antibiotic	10 weeks	21 days	Feed	110 g per kg of feed	$0.26	$0.83
6	Tylosin	Antibiotic	10 weeks	21 days 21 days	Feed Feed	110 g per kg of feed 44 g per kg of feed	$0.26	$1.20
7	Tylosin	Antibiotic	10 weeks	7 days 7 days	Water Feed	83 mg per l of water 110 g per kg of feed	$0.41 $0.26	$1.03
8	Tylosin	Antibiotic	10 weeks	14 days	Water	83 mg per l of water	$0.41	$1.25
9	Tiamulin	Antibiotic	10 weeks	14 days	Feed	121.4 g per 1,000 kg of feed	$0.10	$0.21
10	Lincomycin	Antibiotic	10 weeks	21 days	Feed	110 kg per 1,000 kg of feed	$0.19	$0.63
11	Tylvalosin	Antibiotic	10 weeks	14 days	Feed	42.5 g per 1,000 kg of feed	$0.62	$0.46
12	Tylvalosin	Antibiotic	10 weeks	5 days	Water	50 mg per l of water	$0.66	$1.04

Enterisol Ileitis and Porcilis Ileitis are the vaccines available for managing *L. intracellularis* in pig herds. Enterisol Ileitis is a live vaccine that can be administered via a drench right at weaning. Enterisol Ileitis has been available to producers for more than 10 years while Porcilis Ileitis is a newer option. Unlike Enterisol Ileitis, Porcilis Ileitis is a bacterin, and is assumed to be given as an injection right at weaning in the nursery. While there is debate as to when it is best to provide vaccines to piglets, the convenience of vaccinating when piglets are being handled at weaning often dictates when they are provided.

The remaining management options for *L. intracellularis* are antibiotics to be used for treating the disease once clinical signs appear. Four of these options are variations of administering tylosin, either in feed or water, or a combination of both. Tiamulin can be provided as a feed additive, lincomycin can be provided as a feed additive and tylvalosin can be administered via feed or water. Given that treatment must occur after clinical signs appear, this model assumes that each of these treatment options start to be administered 1 week after clinical signs appear (11 weeks of age). Therefore, treatment begins in the last week in the nursery barn and may continue into the finishing barn.

### Efficacy of Management Options

The 12 disease management options can improve the production performance of a sick pig with the extent of the improvement depending on the option and the severity of the with *L. intracellularis* outbreak. The improvements in average daily gain, decrease in feed efficiency and decrease in mortality for each option under each of the three disease scenarios are summarized in Table 5.

**Table 5:** Change in production parameters from management options for *L. intracellularis*.

		***L. intracellularis*** **outbreak scenario**		
		**Worst-case (%)**	**Expected (%)**	**Best-case (%)**
**PREVENTATIVE MANAGEMENT**				
Chlortetracycline^a^ (in-feed, 100 ppm, 2 weeks)	Avg. daily gain	183	163	142
	Feed efficiency	79	85	90
	Mortality	50	62	75
Tylosin^b^ (in-feed, 110 ppm, 3 weeks)	Avg. daily gain	191	168	146
	Feed efficiency	67	75	83
	Mortality	93	95	97
Enterisol Ileitis^c^ (Drench)	Avg. daily gain	117	113	108
	Feed efficiency	90	93	95
	Mortality	65	73	82
Porcilis Ileitis^d^ (injection)	Avg. daily gain	106	104	103
	Feed efficiency	92	94	96
	Mortality	49	62	74
**TREATMENT MANAGEMENT**				
Tylosin^e^^,^ ^f^ (in-feed, 110 ppm, 2 weeks)	Avg. daily gain	120	115	110
	Feed efficiency	86	90	93
	Mortality	50	63	75
Tylosin^e^^,^ ^f^ (in-feed, 110 ppm, 3 weeks, then in-feed, 44 ppm, 3 weeks)	Avg. daily gain	120	115	110
	Feed efficiency	86	90	93
	Mortality	50	63	75
Tylosin^e^^,^ ^f^^,^ ^g^ (in-water, 83 mg/l, 1 week, then in-feed, 110 ppm, 1 week)	Avg. daily gain	158	144	129
	Feed efficiency	71	78	85
	Mortality	53	65	77
Tylosin^g^ (in-water, 83 mg/l, 2 weeks)	Avg. daily gain	196	172	148
	Feed efficiency	55	66	77
	Mortality	56	67	78
Tiamulin^h^ (in-feed, 100 ppm, 2 weeks)	Avg. daily gain	129	122	115
	Feed efficiency	86	89	93
	Mortality	0	25	50
Lincomycin^i^ (in-feed, 100 ppm, 3 weeks)	Avg. daily gain	123	117	11
	Feed efficiency	81	86	91
	Mortality	62	71	81
Tylvalosin^f^^,^ ^k^ (in-feed, 42.5 ppm, 2 weeks)	Avg. daily gain	120	115	110
	Feed efficiency	86	89	93
	Mortality	91	93	96
Tylvalosin^k^ (in-water, 50 ppm, 5 days)	Avg. daily gain	155	141	127
	Feed efficiency	76	82	88
	Mortality	0	25	50

a McOrist et al. (18)^*^.

b McKay et al. (19)^*^.

c *McOrist and Smits. (11)*.

d *O'Brien et al. (20)*.

e *Veenhuizen et al. (21)*.

f *Pommier et al. (22)*.

g Paradis et al. (23)^*^.

h Walter et al. (24)^*^.

i Winkelman (15)^*^.

j Guedes et al. (25)^*^.

k United States Department of Food and Drug Administration (26)^*^.

The values given in Table 5 are based on a variety of studies rather than from a single coordinated research program on *L. intracellularis* and its management strategies. Sources for Table 5 with an Asterix involved clinical trials in which the treatment group were given a vaccine/antibiotic while the control group was not. Both groups were inoculated with *L. intracellularis* and their production performance measured over a set period of time. Because all pigs were inoculated with *L. intracellularis*, the results of these studies are assumed to show the benefits of vaccines/antibiotics for managing a worst-case incidence. This data was then used to make assumptions about the impact of the vaccine/antibiotic for managing an expected *L. intracellularis* incidence (25% less beneficial) and a best-case *L. intracellularis* incidence (50% less beneficial). Citations in Table 5 without an Asterix involved naturally infecting the pigs with *L. intracellularis* rather than inoculating them. The results of these studies are assumed to show the benefits of vaccines/antibiotics for managing an expected incidence. Data from these studies were used to make assumptions about the worst-case scenario (33% more beneficial) and the best-case scenario (33% less beneficial). For any missing data, assumptions were made by comparing between management strategies to fill in the gaps.

The values in this table reflect the change in the pig's performance compared to when the disease is not being managed. For example, the average daily gain for a 10-week-old pig in a worst-case disease scenario is 0.53 kg. If preventative chlortetracycline had been used, we multiply 0.53 kg by 1.83 to get an average daily gain 0.97 kg. However, a 10-week-old pig that is healthy only has an average daily gain of 0.66 kg. In this model, a pig's production improvement from management is limited to the production of the healthy pig so its average daily gain would improve from 0.53 to 0.66 kg. The values in Table 5 for feed conversion and mortality are often <100%, as a lower feed conversion value and mortality value indicate improved production performance.

## Results

### Financial Impacts of *L. intracellularis*

The net return to the farrow-to-finish pig farm under baseline productivity assumptions in which pigs reach 135.87 kg during their 27 weeks of growth is $4.99 per pig or $115,090 annually. *L. intracellularis* decreases farm profits by reducing the average daily gain, decreasing the feed efficiency and increasing the mortality on average for the entire growing herd (15, 27). Reducing average daily gain increases the amount of time that it takes for a pig to reach market weight. Due to space limitations in a farrow-to-finish operation, shipping schedules are typically followed in order to make room for the next group of pigs that are coming into the barn, so the market pigs shipped are lighter and less desirable for the processor. Reduced feed efficiency of the growing pigs means more feed is required, and thus higher costs per pound of weight gain. Increased mortality reduces the number of market hogs for sale, but also means that the fixed costs for the farm must now be spread across fewer pigs, thus increasing the total cost to raise each pig.

In the best-case scenario in which the impacts of *L. intracellularis* are minimized, the pig's average daily gain and feed efficiency both fall by 6% for weeks 10 and 11 and morality in the nursery barn increases to 4%. The productivity reduction leads to a 0.55 kg decrease in shipping weight, 5 fewer pigs being shipped per week and an increase in fixed costs by $0.24 per pig. With revenue per pig decreasing to $183.08 and the cost per pig increasing to $179.27, the net return per pig falls 24% from $4.99 in the baseline scenario to $3.81. As a result, total annual net returns fall from $115,090 to $86,869 under the best-case disease scenario.

The expected effects from *L. intracellularis* are a fall in average daily gain by 13%, a decrease in feed efficiency by 15% for a 5-week period, and an increase in nursery mortality from 3 to 6%. As a result, 14 fewer pigs are shipped per week compared to the baseline scenario. For pigs that are shipped, their average weight decreases by 3.10 kg and the fixed cost per pig increases by $0.74. These lighter pigs only bring in $179.64 per animal and the cost per pig increases to $180.24 resulting in the net return per pig falling from $4.99 in the baseline scenario to $-0.60. Thus, the financial impact of *L. intracellularis* is almost $130,000 as annual net returns fall from $115,090.39 to a loss of –$13,452.49.

In the worst-case scenario of a *L. intracellularis* outbreak, average daily gain falls 20%, feed efficiency drops 25% for weeks 10–18, and nursery mortality increases from 3 to 8%. The result is 24 fewer pigs are shipped per week and the pigs that are shipped are 9.72 kg lighter. With fixed costs increasing by $1.26 per pig, the total cost per pig increases to $181.21, while the revenue falls to $170.68 per pig. Therefore, the net return per pig falls from $4.99 in the baseline scenario to a loss of $10.53 and total annual net returns fall by $345,300. A summary of the financial impacts from farm under these three scenarios for *L. intracellularis* is given in Table 6.

**Table 6:** Financial Impact of *Lawsonia intracellularis* per marketed hog on a farrow-to-finish swine farm with 1,000 sows.

	**Healthy**	***L. intracellularis*** **outbreak scenario**		
	**Scenario**	**Worst-case**	**Expected**	**Best-case**
Revenue/marketed pig	$183.83	$170.68	$179.64	$183.08
**COSTS**				
Feed costs	$115.33	$116.37	$115.95	$115.51
Variable costs	$40.35	$40.44	$40.40	$40.37
Fixed costs	$23.15	$24.41	$23.89	$23.39
Cost/marketed pig	$178.83	$181.21	$180.24	$179.27
Profit/marketed pig	$\underline{\underline{\$4.99}}$	$\underline{\underline{–\$10.53}}$	$\underline{\underline{–\$0.60}}$	$\underline{\underline{\$3.81}}$
Weekly profit	$2,213.28	–$4,427.12	–$258.70	$1,670.56
Annual profit	$115,090.39	–$230,209.99	–$13,452.49	$86,869.01

*The single lines were included to show a subtraction or addition was occurring*.

*The double lines were to show the end of “per pig” metrics and movement to the farm level measurements*.

### Cost-Effectiveness of Management Options

The estimated monetary benefits of each antibiotic and vaccine management strategy to deal with the three scenarios of a *L. intracellularis* outbreak are listed in Table 7. The profit levels are compared to the healthy baseline scenario and the corresponding disease scenario. By definition, the presence of *L. intracellularis* even with a management option reduces farm returns compared to the scenario with no *L. intracellularis*. The cost-effectiveness of the options managing the disease are determined by comparing the returns with treatment compared to profits without any treatment for the disease. In addition to the net benefits of the option, which are in some cases negative, suggesting the cost of the option are greater than the reduction in the disease impacts, the cost-effective ranking of the treatment strategies is provided for each of the three disease scenarios.

**Table 7:** Cost-effectiveness and ranking of management options for *L. intracellularis*.

		***L. intracellularis*** **outbreak scenario**					
	**Financial measure**	**Worst-case**	**Rank**	**Expected**	**Rank**	**Best-case**	**Rank**
**PREVENTATIVE MANAGEMENT**							
Chlortetracycline	Profit per pig	$3.93	1	$4.30	1	$4.73	1
	$ **Δ** from healthy base	–$25,402		–$16,770		–$6,067	
	$ **Δ** from disease base	$319,897		$111,772		$22,153	
Tylosin	Profit per pig	$2.50	3	$3.49	3	$4.35	3
	$ **Δ** from healthy base	–$60,166		–$37,021		–$15,754	
	$ **Δ** from disease base	$285,134		$91,521		$12,466	
Enterisol Ileitis	Profit per pig	–$4.08	11	$1.34	11	$3.37	12
	$ **Δ** from healthy base	–$207,035		–$84,617		–$37,718	
	$ **Δ** from disease base	$138,264		$43,925		–$9,497	
Porcilis Ileitis	Profit per pig	–$6.15	12	$0.46	12	$3.23	12
	$ **Δ** from healthy base	–$255,417		–$104,604		–$40,712	
	$ **Δ** from disease base	$89,882		$23,938		–$12,490	
**TREATMENT MANAGEMENT**							
Tylosin	Profit per pig	–$1.12	8	$2.57	7	$3.79	5
	$ **Δ** from healthy base	–$140,529		–$56,239		–$27,725	
	$ **Δ** from disease base	$204,770		$73,302		$495	
Tylosin	Profit per pig	–$1.50	9	$2.19	9	$3.41	9
	$ **Δ** from healthy base	–$149,228		–$64,958		–$36,503	
	$ **Δ** from disease base	$196,071		$63,584		–$8,282	
Tylosin	Profit per pig	$2.12	4	$2.72	6	$3.55	8
	$ **Δ** from healthy base	–$66,879		–$52,979		–$33,402	
	$ **Δ** from disease base	$278,420		$75,563		–$5,180	
Tylosin	Profit per pig	$1.78	5	$2.44	8	$3.30	11
	$ **Δ** from healthy base	–$66,879		–$59,558		–$39,184	
	$ **Δ** from disease base	$278,420		$68,984		–$10,962	
Tiamulin	Profit per pig	$1.24	6	$3.63	2	$4.42	2
	$ **Δ** from healthy base	–$86,632		–$31,524		–$13,273	
	$ **Δ** from disease base	$258,667		$97,018		$14,948	
Lincomycin	Profit per pig	$1.12	7	$2.96	5	$3.89	4
	$ **Δ** from healthy base	–$89,762		–$47,785		–$25,649	
	$ **Δ** from disease base	$225,537		$80,757		$2,572	
Tylvalosin	Profit per pig	–$2.06	10	$2.16	10	$3.798	6
	$ **Δ** from healthy base	–$160,412		–$66,552		–$28,471	
	$ **Δ** from disease base	$184,888		$61,990		–$249	
Tylvalosin	Profit per pig	$2.67	2	$3.11	4	$3.56	8
	$ **Δ** from healthy base	–$53,460		–$43,411		–$33,063	
	$ **Δ** from disease base	$291,839		$85,131		–$4,841	

In the best-case *L. intracellularis* scenario, chlortetracycline is the most profitable disease management option of the 12 listed. At a cost of $0.25 per pig, this pre-emptive medication reduces the production impacts of *L. intracellularis* upon infection. When compared to no management, administering chlortetracycline increases the net return per pig by $0.92 and increases annual net farm returns from $86,869.01 to $109,022.52. This is only a $6,067.88 fall in annual profits from the healthy, baseline scenario. Of the 12 management options, seven of them have lower net returns than non-intervention in the best-case disease scenario. However, in order of cost-effectiveness, administering tiamulin, pre-emptive tylosin, lincomycin, or in-feed tylosin for treatment (110 ppm) have greater net returns than non-intervention. Porcilis Ileitis is the least cost-effective management option, coming at a cost of $1.38 per pig and a net return per pig of $-0.58. If the producer administers Porcilis Ileitis, it results in $12,490.80 less profit per year for the farm than doing nothing. Therefore, with chlortetracycline providing a $22,153.51 increase in annual net returns and Porcilis Ileitis providing a –$12,490.80 loss, there is a spread of $34,644.30 in annual net returns between the different options for managing a best-case *L. intracellularis* disease scenario.

In the expected *L. intracellularis* scenario, using any of the 12 management options is more cost-effective for the farm than doing nothing. Again, chlortetracycline is the most cost-effective disease management option available. When compared to non-intervention, administering chlortetracycline increases the net return per pig by $4.90 and increases annual net returns from $-13,452.49 to $98,320.31. This is a net benefit of $111,772.80 and is only $16,770.09 less than annual net returns under the healthy, baseline scenario. Similar to the best-case scenario, Porcilis Ileitis is the least cost-effective management option. However, in the expected disease scenario, the net return remains positive at $0.46 per pig shipped. This amounts to annual farm net returns of $10,486.11 and a $23,938.60 improvement from annual net returns of $-13,4452.49 without intervention. Under the expected scenario, the range in annual net returns for different management options is $87,834.20, with chlortetracycline ($98,320.31) being the highest and Porcilis Ileitis ($10,486.11) being the lowest. One notable change in the rank of management options from the best-case to the expected disease scenario is tylvalosin administered in water. Under the best-case scenario, tylvalosin in water is the 9th ranked management option in terms of cost-effectiveness, but, in the expected scenario, it is ranked 5th. This is because administering tylvalosin in water is expensive, but under the more severe disease scenario, the significant productivity improvements can now be realized.

In the worst-case scenario, without any intervention, the farmer loses $10.53 per pig or $230,209.99 per year. Employing any of the disease management options is more cost-effective than doing nothing, with chlortetracycline being the most ($89,687.78) and Porcilis Ileitis being the least ($-140,327.04) cost-effective. Of the 12 management options, seven of them will keep net returns above zero under the worst-case disease scenario, while five of them result in an annual net loss in income. In addition to Porcilis Ileitis, these products include Enterisol Ileitis ($-91,945.60), tylvalosin in feed ($-45,321.86), tylosin in feed at 110 and 44 ppm ($-34,138.45) and tylosin in feed at 110 ppm ($-25,439.43). With net returns being $89,687.78 with chlortetracycline and $-140,327.04 for Porcilis Ileitis, the spread between management options is quite significant under the worst-case disease scenario at $230,014.82 per year.

### Sensitivity Analysis

A sensitivity analysis was performed on the efficacy of each of the 12 management options in all three of the disease scenarios. This analysis compared the rankings of each management option when their efficacy in managing mortality, average daily gain and feed efficiency were either increased or decreased by 10%. In general, the results of this sensitivity analysis suggest that the results of this model are robust. The rankings of management options do not change significantly when their efficacy is either increased or decreased by 10% for each of the three productivity variables tested. Prophylactic antibiotics remain as two of the most cost-effective options, while tiamulin and tylvalosin are the more cost-effective metaphylactic options. Furthermore, the vaccines remain as two of the least cost-effective options in each of the scenarios run.

## Discussion

As livestock producers transition to reduced antibiotic use, there will be significant discussion between industry representatives and healthcare experts about the best way to execute this transition. An effective plan will consider the value that antibiotics provide to farmers while recognizing the significant impact of use on antibiotic resistance. While there has been significant research on the risks of resistance in humans and animals (the costs of antibiotic use), there is little information on the benefits that specific antibiotics provide for managing livestock diseases. This research examined the production and financial impacts of using alternative management strategies for treating *L. intracellularis* in pigs and, in the process, has also provided a framework for assessing the net value of alternative antibiotics for different livestock and different diseases.

These scenarios show the net financial benefits of different antibiotics and vaccines for managing *L. intracellularis*. Without any treatment, a *L. intracellularis* outbreak would reduce net farm income from $115,090 to $88,869 under the best-case scenario and to a loss of $230,210 under a worst-case scenario. The small changes in growth rate, feed efficiency, and mortality that are impacted by the disease can have a significant impact on the financial success of a farm and highlight the costs of a complete ban of antibiotic use.

For most of the 36 management scenarios, it was more cost-effective to intervene rather than do nothing when facing a *L. intracellularis* outbreak. This was certainly true for the more severe disease scenarios, as the substantial productivity improvements warranted the added cost of the medication. However, for each of the three disease scenarios, there was a significant difference in net returns between the most beneficial and least beneficial options.

In all three of the disease scenarios, prophylactic medication of in-feed chlortetracycline was the most profitable option and prophylactic in-feed tylosin was the third most profitable option for managing *L. intracellularis*. Part of this can be explained by fact that these medications are provided to the pigs at a much younger age. Medicating younger piglets is much cheaper than medicating older pigs because the younger piglets are smaller and require less medication. Furthermore, when medication is provided prophylactically, it is able to act on the disease as soon as it appears. This contrasts with the treatment scenario, where a farmer or veterinarian must first notice the clinical signs before the pigs are medicated. This means that there is a period of time where the disease is able to exist, multiply, and impact the pigs before they finally receive the medication they need. In the best-case and expected disease scenario, the second most cost-effective management option was tiamulin. In the worst-case scenario, the second most cost-effective management option was tylvalosin in water. Despite being provided to older pigs, tiamulin is very affordable and provides modest improvements in productivity. This makes it a good candidate for managing less severe *L. intracellularis* incidences. Tylvalosin in water is much more expensive but provides significant improvements in average daily gain, feed efficiency and mortality. Therefore, in the worst-case disease scenario, the increased cost is justified to mitigate the more severe production problems.

With Canada's livestock industries transitioning to reduced antibiotic use, it is likely that farmers will be required to reduce or eliminate prophylactic antibiotic administration in favor of using antibiotics metaphylactically or for individual treatment. This prevention vs. treatment argument is significant with respect to reducing antibiotic use as this model shows that there are significant financial benefits to managing diseases prophylactically. Furthermore, prophylactic antibiotic administration is likely to improve animal welfare as the livestock's production problems are either avoided entirely or managed more quickly. However, increased antibiotic use as a result of prophylactic administration is seen as a significant contributor to antibiotic resistance for reasons explained previously. Policy on antibiotic use needs to balance the risks of resistance and corresponding threat to human health along with animal welfare implications of untreated diseased animals (28), which is not considered in this study, with the financial benefits to farmers from prophylactic antibiotics, which is estimated in this study. In the absence of an understanding of the farm level impacts, costs on the farm of policy interventions may well outweigh the public health benefits.

The modeling framework can be used to determine the minimum price required by farmers who raise livestock for niche markets, including “raised without antibiotics” (RWA). Based on this study, we can see from Table 7 that for a RWA farmer to make the same as a conventional farmer they would need $0.92 per pig in the best-case scenario, $2.96 per pig in the expected scenario and $8.01 per pig in the worst-case scenario. Although similar negative financial impacts of RWA were also estimated for dealing with endemic PRRSV in swine herds by Dee et al. (29), the extent of the net financial impacts will vary depending on farm and market conditions that can be simulated with the model developed for this study. Singer et al. (28) find that the switch to RWA by livestock producers has been dictated by others further along the supply chain and these financial estimates provide the higher price required by producers to compensate for changing disease management systems.

In addition to the way that antibiotics are used, the type of antibiotic used is an important consideration for human health risk. As previously stated, the World Health Organization groups antibiotics into four classes depending on their relative importance to human health. Class 4 is the least important and Class 1 is the most important. The results of this study suggest that chlortetracycline is the most cost-effective option for managing *L. intracellularis* in all three disease scenarios. Chlortetracycline is a Class 3 antibiotic and is therefore less important to human health than a Class 2 antibiotic or Class 1 antibiotic, like tylosin. Tiamulin, the second most cost-effective option for the best-case and expected disease scenarios, is a pleuromutilin and Class 4 antibiotic. Tylvalosin is the second most cost-effective management option for the worst-case scenario, but is a macrolide and a Class 1 antibiotic. Therefore, if prophylactic antibiotic administration were allowed, it would be best to manage *L. intracellularis* prophylactically with chlortetracycline. In addition to being the most cost-effective option, chlortetracycline is only a Class 3 antibiotic and presents a low risk to human health. If clinical signs appeared after using chlortetracycline, they would likely be less severe, and the farmer could handle them by administering tiamulin. Similar to chlortetracycline, tiamulin is a cost-effective strategy and, as a Class 4 antibiotic, it is the least important for human health. Taking this approach would leave tylvalosin, one of the most important antibiotics (Class 1), for emergency situations with very severe *L. intracellularis*-related production problems. Based on cost-effectiveness, if prophylactic antibiotics become banned, tiamulin is the only management option that the farmer can use to avoid a severe *L. intracellularis* incidence. Therefore, this policy would increase the chance of the farmer having to use a Class 1 antibiotic, like tylvalosin, which is more important to human health. This analysis highlights how farm production and financial data can be paired with information about human health risk to evaluate antibiotic usage and policy.

To our knowledge this is the first study that takes a comprehensive bioeconomic approach to estimating the net financial benefit of each antibiotic and each vaccine labeled for managing a specific disease in pigs. By calculating the cost-effectiveness of these management strategies for different disease severity levels, it can be seen which intervention strategies provide the greatest benefit for producers who are facing this illness. This model uses meta-data from the literature to specify parameters to generate results. For the model to produce more accurate results, a clinical study should be conducted with the intent of providing the values for these parameters. This would allow for more opportunity to examine the range of effectiveness for each disease management option. While the model in this study used herd-level averages to measure the production impact of *L. intracellularis*, it can be easily modified to include multiple sub-groups within the herd. This would more accurately reflect a true disease scenario, where only some pigs are infected, and some have more severe production problems than others. However, the intricacies of these types of disease scenarios can become quite complicated, so a straightforward model limits the number of required assumptions. In addition, the model could be extended to include additional preventative strategies, such as biosecurity measures, to assess the cost-effective means for preventing, rather than managing a disease. For veterinarians or farmers that know their specific herd scenario, a more intricate model may be more useful as a decision-making tool for different disease management options. The framework of this model can be extended to reflect different business structures, such as a “loop” where different farmers specialize in raising pigs through specific life stages. Farmers focusing on one area of production have different incentives than a farmer who manages a farrow to finish operation and thus different reasons for changing reduce antibiotic use.

Irrespective of the assumptions made or the potential applications of this model, this research provides a framework that can perform a coordinated evaluation of the value that antibiotics provide to the swine industry and other livestock industries. If this type of study were repeated (with enhanced parameter values) for other diseases and other livestock, a representative assessment could be made as to which antibiotics provide the greatest benefit and which provide the least amount of benefit to each of the livestock industries. Then, through work with public health officials and other experts in antibiotic resistance, a strategic plan could be made for reducing antibiotic use. This plan would value the importance of antibiotics in maintaining the financial viability of farms, while considering the significant risk that the use of some antibiotics presents to human and animal healthcare systems.

## Conclusion

Antibiotics and vaccines play significant roles in helping farms remain profitable in the presence of a disease. In this model, the calculated net return of a farrow-to-finish operation with 1,000 sows was $115,090.39 annually. In the best-case, expected and worst-case disease scenarios for *L. intracellularis*, annual net returns without intervention were $86,869.01, –$13,452.49, and –$230,209.99, respectively. Prophylactic chlortetracycline was the most cost-effective management option in each of the three scenarios with an annual net return of $109,022.52, $98,320.31, and $89,687.78 in the best-case, expected and worst-case disease scenarios, respectively. The Porcilis Ileitis vaccine was the least cost-effective management strategy in each scenario. Although vaccines are emerging as viable alternatives to prophylactic antibiotic use, this study suggests that vaccines result in significant financial losses for the farmer. Ceasing prophylactic antibiotic use may, unintendedly, lead to an increase in the use of antibiotics that are more important for human health. If prophylactic chlortetracycline (Class 3 antibiotic), were to be banned in order to reduce total antibiotic use, there may be a greater chance of more severe *L. intracellularis*. In this case, farmers might need to use antibiotics that are more important to human health like tylosin or tylvalosin (both Class 1 antibiotics) to handle the disease. Although antibiotic resistance is complex, discussions about reducing antibiotic resistance should acknowledge the risk and the benefit that comes from specific livestock antibiotic use. A coordinated research program that pairs the framework in this study with field trials will help to inform this discussion which will lead to a more comprehensive plan for reducing antibiotic resistance caused by livestock antibiotic use.

## Data Availability Statement

The datasets generated for this study are available on request to the corresponding author.

## Author Contributions

TJ gathered the data, conducted the analysis, and wrote the initial draft. AW organized and supervised the research and did final editing. MM helped in the simulation model. ZP provided expertise on the disease modeling and cost estimates.

### Conflict of Interest

The authors declare that the research was conducted in the absence of any commercial or financial relationships that could be construed as a potential conflict of interest.
